# Supplementary material for: Metabolite profiling from the fermentation of marine-derived extracts by Lactobacillus acidophilus LB
Source: PeerJ. 2025 Dec 3;13:e20399. doi: 10.7717/peerj.20399 (PMC12681230; doi:10.7717/peerj.20399)
Supplement: Supplemental Information 1 [file peerj-13-20399-s001.docx]

Supplementary 1. Sample preparation

|  | **Sample** | **Sample labeling** | |
| --- | --- | --- | --- |
| 1 | Medium containing Spirulina (SPi) | Supernatant | S.SPi |
|  |  | Centrifugal pellet | B.SPi |
|  | *L. acidophilus* LB grown in medium (SPi-LBA) | Supernatant | S.SPi-LBA |
|  |  | Centrifugal pellet | B.SPi-LBA |
| 2 | Medium containing seaweed *Ulva reticulata* (Ulva) | Supernatant | S.Ulva |
|  |  | Centrifugal pellet | B.Ulva |
|  | *L. acidophilus* LB grown in the medium (*Ulva-*LB*A*) | Supernatant | S.Ulva-LBA |
|  |  | Centrifugal pellet | B.Ulva-LBA |
| 3 | Medium containing seaweed *Caulerpa lentillifera* (Caulerpa) | Supernatant | S.Caulerpa |
|  |  | Centrifugal pellet | B.Caulerpa |
|  | *L. acidophilus* LB grown in the medium (*Caulerpa-*LB*A*) | Supernatant | S.Caulerpa-LBA |
|  |  | Centrifugal pellet | B.Caulerpa-LBA |
| 4 | *L. acidophilus* LB grown in MRS medium (*MRS-*LB*A*) | Supernatant | S.MRS-LBA |
|  |  | Centrifugal pellet | B.MRS-LBA |
